# Gene expression changes linked to antimicrobial resistance, oxidative stress, iron depletion and retained motility are observed when *Burkholderia cenocepacia *grows in cystic fibrosis sputum

**DOI:** 10.1186/1471-2334-8-121

**Published:** 2008-09-19

**Authors:** Pavel Drevinek, Matthew TG Holden, Zhaoping Ge, Andrew M Jones, Ian Ketchell, Ryan T Gill, Eshwar Mahenthiralingam

**Affiliations:** 1 Cardiff School of Biosciences, Cardiff University, Cardiff, UK; 2 The Wellcome Trust Sanger Institute, Cambridge, UK; 3 Center for Bioinformatics, University of North Carolina, Chapel Hill, NC, USA; 4 Bradbury Cystic Fibrosis Unit, Wythenshawe Hospital, Manchester, UK; 5 Cardiff Adult Cystic Fibrosis Centre, Llandough Hospital, Penarth, UK; 6 Department of Chemical and Biological Engineering, University of Colorado, Boulder, CO, USA

## Abstract

**Background:**

Bacteria from the *Burkholderia cepacia *complex (Bcc) are the only group of cystic fibrosis (CF) respiratory pathogens that may cause death by an invasive infection known as cepacia syndrome. Their large genome (> 7000 genes) and multiple pathways encoding the same putative functions make virulence factor identification difficult in these bacteria.

**Methods:**

A novel microarray was designed to the genome of *Burkholderia cenocepacia *J2315 and transcriptomics used to identify genes that were differentially regulated when the pathogen was grown in a CF sputum-based infection model. Sputum samples from CF individuals infected with the same *B. cenocepacia *strain as genome isolate were used, hence, other than a dilution into a minimal growth medium (used as the control condition), no further treatment of the sputum was carried out.

**Results:**

A total of 723 coding sequences were significantly altered, with 287 upregulated and 436 downregulated; the microarray-observed expression was validated by quantitative PCR on five selected genes. *B. cenocepacia *genes with putative functions in antimicrobial resistance, iron uptake, protection against reactive oxygen and nitrogen species, secretion and motility were among the most altered in sputum. Novel upregulated genes included: a transmembrane ferric reductase (BCAL0270) implicated in iron metabolism, a novel protease (BCAL0849) that may play a role in host tissue destruction, an organic hydroperoxide resistance gene (BCAM2753), an oxidoreductase (BCAL1107) and a nitrite/sulfite reductase (BCAM1676) that may play roles in resistance to the host defenses. The assumptions of growth under iron-depletion and oxidative stress formulated from the microarray data were tested and confirmed by independent growth of *B. cenocepacia *under each respective environmental condition.

**Conclusion:**

Overall, our first full transcriptomic analysis of *B. cenocepacia *demonstrated the pathogen alters expression of over 10% of the 7176 genes within its genome when it grows in CF sputum. Novel genetic pathways involved in responses to antimicrobial resistance, oxidative stress, and iron metabolism were revealed by the microarray analysis. Virulence factors such as the cable pilus and Cenocepacia Pathogenicity Island were unaltered in expression. However, *B. cenocepacia *sustained or increased expression of motility-associated genes in sputum, maintaining a potentially invasive phenotype associated with cepacia syndrome.

## Background

Cystic fibrosis (CF) is the most common hereditary disease in Caucasian population affecting one in every 2,500 – 4,500 newborns. People with CF lack a functional chloride transporter normally present in epithelial cell membranes which results in multiple organ system impairment. The respiratory tract is one of the most profoundly affected systems where the defect in ion transport causes production of highly viscous mucus. The resulting ineffective mucocilliary clearance in the lung leads to colonization of the airway with several bacterial pathogens and ultimately it is respiratory infections that are the major cause of morbidity and mortality in individuals with CF. The most problematic bacterial infections are caused by *Pseudomonas aeruginosa *and the *Burkholderia cepacia *complex (Bcc), and are characterized by low responsiveness to antibiotic therapy and significant reductions in a patient's lung function. Both bacteria also pose the risk of epidemic spread within the CF community [1,2], with the Bcc being distributed among CF patients to much smaller extent (3 – 30%; [3] compared to *P. aeruginosa *70 – 80%; [1]). However, the clinical relevance of Bcc infection is underlined by the fact that it is the only known CF pathogen which can enter the blood circulation and lead to fulminating septicaemia with acute respiratory failure called "cepacia syndrome" [2]. The Bcc represents a broad collection of closely related bacterial species, of which *Burkholderia cenocepacia *and *Burkholderia multivorans *are the most dominant species in CF infection [1].

Although rapid clinical decline and epidemic spread is linked to infection with a number of Bcc species, *B. cenocepacia *is regarded as one of the most virulent and problematic CF pathogens [2]. This species accounts for 50 – 90% of all Bcc infections diagnosed in CF patients in different national CF centres [1,4] and includes strains with a well documented history of patient-to-patient transmission, as well as strains exhibiting a high virulence potential [5,6]. Several epidemic *B. cenocepacia *strains have been described [7]. One strain type, known as ET12, was one of the first highly transmissible strains to be described and caused multiple infection outbreaks in Canada and the United Kingdom that were associated with significant mortality among CF patients [8]. As a result of its clinical significance, the ET12 *B. cenocepacia *strain J2315 [7] was the first Bcc pathogen selected for genome sequence determination at the Wellcome Trust Sanger Institute. The complete J2315 genome subsequently became the basis for developing the first custom microarray of a *B. cenocepacia *species [9,10].

A number of pathogenic traits have been identified in *B. cenocepacia *including lipopolysaccharide, various adhesins, flagella, siderophores, type III secretion system, quorum sensing, genomic islands, haemolysins, extracellular proteases, exopolysaccharide, resistance to antibiotics and to reactive oxygen species, and the ability to form biofilms (reviewed in [2]). Despite this extensive research, the individual contribution of each virulence determinant and their potential interaction with other virulence factors during CF infection has not been extensively addressed. In addition, *Burkholderia *genomes are among the largest in bacteria ranging from 6 Mb to greater 9 Mb [11]. Their genomes also contain multiple pathways for related functions and considerable gene redundancy due to presence of paralogous genes [12]. Hence, pin-pointing which genes are active in specific environments is not straightforward due to the large number of predicted functional redundancies in their genomes.

In order to characterize the molecular processes that take place in *B. cenocepacia *at an early stage of infection, we studied the global gene expression changes that occur during growth in CF sputum modelled under laboratory conditions. By using a genomic microarray approach, we were also able to profile expression and uncover which of the many paralogous gene systems in *B. cenocepacia *are active in an environment mimicking infection. Examining gene expression in sputum-based models has only been performed with *P. aeruginosa*, using UV-irradiated CF sputum [13] or artificial sputum [14]. In this first complete study of *B. cenocepacia *sputum-transcriptomics, we were able to work with CF sputum samples in a non-sterilized, close to native form since the CF individuals examined were chronically infected with ET12 strains that were determined by microbial reference centres using standard procedures [1,2] to be genetically identical to our experimental strain J2315. The transcriptome of a *B. cenocepacia *population resulting directly from the pathogen-host interaction in sputum from three CF individuals was measured using multiple biological replicates. A complex picture of the pathophysiological state of *B. cenocepacia *during an early stage of infection was developed by interpreting the expression levels of individual genes that were specifically activated or suppressed in the CF sputum environment. Pathogenic traits that were altered in this model of growth during respiratory infection included activation of protective mechanisms against antibiotics and reactive oxygen species, a response to balance changes in concentration of internal iron, and upregulation of two well known virulence factors, flagella and metalloproteases.

## Methods

### Microarray

A custom 2 × 11 K microarray for *B. cenocepacia *was developed using Agilent's two colour 60-mer ink jet synthesis platform (Agilent Technologies, Santa Clara, California). A partial genomic microarray and labelling procedures using this technology had already been validated [10]. The microarray was composed of 10,807 probes which were specific to the following features; annotated J2315 CDS (a total 7,251 probes including duplicate probes for several genes), selected J2315 intergenic regions (1,489), probes for CDS specific to other two sequenced *B. cenocepacia *strains AU1054 and HI2424 (1,457), and 610 probes serving as technical controls (the array definition file is deposited in ArrayExpress under the accession number A-MEXP-867). The genome sequence of *B*. cenocepacia strain J2315 was produced by the Pathogen Sequencing Group at the Sanger Institute, Hinxton, Cambridge , and the sequences of strains AU1054 and HI2424 were produced by the produced by the US Department of Energy Joint Genome Institute .

### Sputum samples

The respiratory samples were obtained from adult CF patients as routine diagnostic specimens sent for microbiological analysis. For each patient the clinical condition, bacterial infection and antibiotics administered have been summarised in Table 1. Sputa from three CF patients infected with the ET12 *B. cenocepacia *were used in the microarray based analysis: patient 1 and patient 2 were clinically stable at the time of sampling (sputum 1 and 2), while patient 3 was suffering an exacerbation (sputum 3; Table 1). Subsequently, along with sputum 2 and 3, three additional sputum samples from three further CF patients were examined using quantitative PCR to validate the microarray observed gene expression: patient 4 (sputum 4) was infected with *B. cenocepacia *and clinically stable; patient 5 (sputum 5) was infected only with *P. aeruginosa *and exacerbating, and patient 6 (sputum 6) was also only infected with *P. aeruginosa *but stable at the time the sample was provided (Table 1).

**Table 1:** Sputum samples examined

**Patient (sputum) number:**	**Patient's clinical status:**	**Infection:**	**Antibiotic treatment:**
1	Clinically stable	*B. cenocepacia*	azithromycin, flucloxacillin, aztreonam, tobramycin
2	Clinically stable	*B. cenocepacia*	azithromycin, minocycline
3	Pulmonary exacerbation	*B. cenocepacia*	azithromycin, ceftazidime, gentamicin
4	Clinically stable	*B. cenocepacia*	azithromycin, flucloxacillin
5	Pulmonary exacerbation	*P. aeruginosa*	meropenem, tobramycin
6	Clinically stable	*P. aeruginosa*	ciprofloxacin, tobramycin

Each sputum sample was stored frozen in its native form at -20°C and upon arrival to the research laboratory, they were thawed, diluted in a minimal basal salts medium (BSM) containing 14.3 mM glucose and 0.05% casamino acids [15] to a concentration of 12.5% w/vol, homogenized by passage through a 10 ml syringe 20 × and archived at -20°C. The total iron concentration of the sputum samples and basal salts media was determined using standard atomic adsorption spectrometry at 248.3 nm in an air acetylene flame (Varian SpectrAA-100 running Spectra-100 Version 2 Software). A standard curve was prepared using a spectrosol iron standard (VWR Scientific, UK).

### Growth conditions

All incubations were carried out at 37°C with shaking in an orbital shaker set at 220 rpm. A 3 ml starter culture of J2315 (harvested at mid-log growth phase growth in Luria-Bertani broth; OD_600 nm _0.5) was split into two aliquots and centrifuged (8 minutes, 650 × g). The pellet was resuspended in 3 ml BSM and 1 ml (~1 × 10^8 ^colony forming units; cfu) inoculated into either: (i) 4 ml BSM (control) or, (ii) 4 ml BSM supplemented with homogenized CF sputum at 12.5% w/vol, giving a final sputum concentraton of 10% w/vol in the 5 ml culture volume (test). Incubation was continued until the culture optical density had increased by OD_600 nm _0.6 (an equal increase in culture density rather than an equal time period of incubation). For each experiment this meant that growth started at an OD_600 nm _of between 0.3 and 0.4 and was then harvested at an OD_600 nm _of between 0.9 and 1.0, providing sufficient time for *B. cenocepacia *to adapt is gene expression to the new growth condition. After growth, the cultures were then immediately cooled in liquid nitrogen and briefly centrifuged (1 minute, 160 × g, 4°C) to remove heavy particulate matter. To lyse human cells and enable better purification of bacteria specific RNA, the supernatants were mixed with an equal volume of 0.2% Triton X-100 in BSM, left for 10 minutes on ice, diluted with 5 volumes of BSM and centrifuged to collect the bacteria (5 minutes, 5,000 × g, 4°C). The resulting pellets were snap frozen in liquid nitrogen and stored at -80°C.

### RNA extraction, labelling and hybridization

Total RNA was isolated using RNeasy Mini Kit (Qiagen) according to the manufacturer's instructions including the optional step of homogenization with a QIAshredder spin columns. To increase RNA yield, we performed an additional sonication step at the beginning of RNA isolation (10 × 10 second pulses, each at 5 μm amplitude, with a Sanyo Soniprep 150 Watt sonicator). After the extraction, the RNA was concentrated by using 7.5 M Lithium Chloride (Ambion). Between 4.5 – 15 μg of RNA was used to synthesize and form labelled cDNA with the CyScribe Post-Labelling Kit (an indirect amino-allyl procedure) following the manufacturer's instructions (Amersham). The fluorescent signal of each labelled sample was checked by electrophoretic separation of 1 μl of sample in a mini-agarose gel followed by scanning (GeneTAC GTLS IV Scanner, Genomic Solutions Inc.). Equal intensities of Cy3- and Cy5-labelled cDNA (approximately 0.5 μg of each) were combined and left to hybridize to the microarray at 65°C for 17 hours. The microarray slides were washed according the manufacturer's instructions (Agilent's microarray processing protocol) with initial washes in the solutions containing two different concentrations of SSPE and N-lauroylsarcosine, and a final wash in the Stabilization and Drying Solution. Signal intensities were scanned with the DNA Microarray Scanner (Agilent) and processed with the Feature Extraction v8.1 software (Agilent).

### Microarray experimental design and analysis

For each patient's sputum, growth and RNA extraction was performed as two independent technical replicates. Each technical replicate comprised a direct comparison of paired Cy3-labelled control (growth in BSM) and Cy5-labelled test (growth in sputum) conditions. Biological replicates were generated by combining analysis of the three CF patients' samples. In addition, a self-hybridization control experiment was performed to evaluate and overcome potential gene-specific dye bias. cDNA from all the control samples, in both Cy3- and Cy5-linked forms, were combined and hybridized on two replicate microarrays. The experimental protocols and raw microarray data can be found in ArrayExpress under the accession number E-MEXP-1261.

Gene expression data analysis was performed using GeneSpring GX 7.3 (Agilent) and only features dedicated to J2315 (i.e., 7,251 CDS and 1,489 intergenic regions) were included in the analysis. Microarray data was examined in two ways. Initially, the three patient-specific biological replicates were each tested using Student's one-sample t-test and their changes in gene expression were defined by fold change relative to the gene expression level in self-hybridization assay; this analysis was performed with a two-fold change filter applied and no multiple testing correction or statistical significance filter of p < 0.05 adopted. The second analysis involved applying the same statistical testing to the original, unmodified combined dataset of six technical replicates in order to identify reproducible trends that result from growth of *B. cenocepacia *in sputum. This analysis was performed with the false discovery rate algorithm applied and a statistical filter of p < 0.05 adopted.

### Quantitative PCR

In order to validate microarray results, five genes with identified altered expression in sputum (4 overexpressed: BCAL0270, BCAM2753, BCAL1107 and BCAM1676; and 1 underexpressed: BCAL1165) were examined individually by using either semi-quantitative reverse transcriptase PCR (RT-PCR) or Real Time PCR (RQ-PCR) approaches. PCR primers targeting the test genes are shown in Table 2. The gene *phaC *(BCAL1861), with constant expression in both control and test microarrays, was used as a control for all quantitative PCR work and amplified using previously described primers [16]; Table 2). Irrespective of growth condition, BCAL1861 possessed a raw fluorescent microarray signal lying above the 75th percentile of all genes with unaltered expression (fold change 0.667–1.334; among approximately 2,500 genes). This also demonstrated that the BCAL1861 transcript was highly abundant in all growth conditions. The semi-quantitative method was based on the endpoint analysis of ordinary PCR reactions varying in their number of cycles as described elsewhere [9].

**Table 2:** PCR primers and conditions for quantitative PCR

**Gene**	**Forward and reverse primer (5' – 3')**	**Product size (bp)**	**Tm (°C)**
**Real-time PCR**			

Control gene BCAL1861 (*phaC*)	AGACGGCTTCAAGGTGGT ACACGGTGTTGACCGTCA	470	62/66
BCAL0270	GCGCGAACCCGATCGAATTC GCCCGCATCCACCAGAAGTG	400	66
BCAM2753	GAACCGCAGACGCTGTACTC AAACGCTCGGTGTTGCGGAC	380	62
BCAL1107	GAATCGACGTATCGGCTCGT ATGATGTGCTTCGGGTTCTTG	377	66
BCAM1676	GACGACCTGTTCCTGCTGTG CGGCTCTTCCTGATGACGTG	534	62

**Semi-quantitative PCR**			

BCAL1165	TGACGCTCGGCACCGTTGAC GCGTGGACCTGCTCGATCTC	400	66

More accurate levels of gene expression fold change were obtained from the RQ-PCR analysis where delta delta C_T _or Pfaffl methods [17] were applied. The primers and gene-specific annealing temperature for PCR conditions are provided in Table 2; each PCR reaction for semi-quantitative RT-PCR contained 0.2 mM (each) dNTPs, 2 mM MgCl_2_, 0.5 μM (each) primer and 0.75 U of GoTaq polymerase (Promega, Madison, USA), while ABsolute SYBR Green Mix (Abgene, Epsom, United Kingdom) with MgCl_2 _concentration of 3 mM was used for the RQ-PCR. The amplifications were run on a MJ Research PTC-200 thermal cycler with the option of real-time fluorescence detection (DNA Engine Opticon). The thermal cycling profile consisted of 15 sec at 95°C, 30 sec at annealing temperature (dependent on the gene being analyzed, see Table 2) and 15 sec at 72°C. Furthermore, the RQ-PCR profile included an initial 15-minute incubation at 95°C required for hot start PCR and a melting curve analysis at the end of PCR.

A template for semi-quantitative PCR was prepared by pooling the same RNAs (representing either growth in sputum or growth in BSM) used previously for microarrays. In addition, we generated five more RNAs from additional growth experiments with five other sputum samples (carried out in accordance with the protocol for growth of J2315 in sputum) and four other RNAs which had represented a response of J2315 to following conditions: (i) low iron concentration (no FeSO_4 _in BSM growth medium), (ii) presence of 0.1% H_2_O_2 _in BSM; (iii) presence of 0.15% H_2_O_2_; and (iv) presence of t-butyl hydroperoxide (0.002% in BSM). Extracted RNA was converted in the amount of 0.1 μg into cDNA by using Improm-II Reverse Transcription System (Promega) and 500 ng of cDNA was added into each PCR reaction. To calculate fold change in expression when RQ-PCR method was used, every run comprised cDNAs from test (growth in sputum or other test condition) as well as from control (growth in BSM) where their gene of interest and their reference housekeeping gene *phaC *[16] were examined each in triplicate. The efficiency of PCR for both genes was determined by serial dilutions of control cDNA over the range of two orders of magnitude.

## Results

### Extraction of *B. cenocepacia *RNA from sputum and microarray performance

Initial attempts to extract RNA from bacteria grown in sputum were inconsistent due to components of sputum interfering with the procedure. Therefore a washing procedure was developed which diluted the sputum in a Triton X100 containing buffer (to lyse human cells), gently centrifuged it to remove large particles, and finally forcefully centrifuged to collect bacteria (see Methods and Materials). Once the RNA extraction procedure had been optimised, the transcriptomic profiles of *B. cenocepacia *obtained were excellent. The array-to-array correlation within a technical duplicate from a single patient's sputum pair ranged from 0.965 to 0.985; variation across all 3 patients ranged from 0.894 to 0.956. These high-correlation coefficients indicated that the custom microarrays provided good quality technical performance over the entire microarray experiment. More importantly, the > 89% similarity between biological replicates demonstrated that there was a high degree of relatedness between sputum samples resulting in a rigorous biological dataset that could be mined for gene expression changes. More than 97% (8,533) of J2315-specific features presented on the microarray were eligible for gene expression analysis as they were consistently above the background noise defined by the scanner detection calls.

### Microarray data analysis

Microarray data was examined in two ways as described in Materials and Methods. The analysis based on the three biological replicates demonstrated that 182 genetic features were significantly over-expressed while 296 were down-regulated with the two fold filter applied. The alternative analysis of the entire dataset where the six arrays were handled as six independent entities revealed 950 genetic features to be significantly altered in expression, with 411 up-regulated and 539 down-regulated. A total of 454 genes overlapped both analytical approaches and only 24 genes (1 up- and 23 downregulated) from the three biological replicate-based analysis were not present in the larger dataset from six technical replicates. Since both analyses demonstrated excellent concordance the results were combined, adding the 24 genes from the biological replicate analysis to the 950 genetic features from the six replicates, allowing 974 genetic elements (coding sequences [CDSs], rRNA, tRNA and intergenic regions) with 2 fold or greater alteration in expression to be evaluated (see Additional Files 1 and 2 for complete lists of up- and downregulated genes respectively). The low, 2-fold, threshold for a change in gene expression was selected to minimize the risk of missing false negative results. For example, multidrug efflux pump protein genes may demonstrate expression changes as low as 1.3 fold, yet such a minor change is known to dramatically alter the physiological activity of the pump [18].

Based on the parameters defined above, *B. cenocepacia *growth in sputum compared to growth in minimal salts medium altered the expression of 723 CDSs representing 10% of all CDSs annotated to date in the genome of J2315 (a total of 7,176). Of these CDSs, 287 were upregulated (see Additional File 1) and 436 downregulated (see Additional File 2). In addition, 33 tRNAs, 2 rRNAs and 2 small RNAs were found over-expressed (Additional File 1). The upregulation of tRNAs may have resulted as a direct consequence of *B. cenocepacia *growth in sputum-enriched BSM which effectively made it a nutrient-rich medium.

Interestingly, approximately one quarter (24.2%) of the differentially expressed genes were unique to strain J2315 in comparison to the complete genomes of other *B. cenocepacia *strains (AU1054 and HI2424); specifically 29% of the upregulated CDSs and 21% of the downregulated CDSs were unique to J2315. Also, around 10% of these unique CDSs were found in indel regions rather than in genomic islands region (11.8% of upregulated and 8.0% of downregulated). Although 18% (130/723) of the CDSs with altered expression lacked functional descriptions, the remaining CDSs differentially expressed in the presence of sputum were composed of several functional groups. Major classes of *B. cenocepacia *genes with altered expression in sputum were selected from the microarray dataset for further discussion and analysis, including genes playing a role in virulence, antimicrobial resistance, iron uptake, protection against reactive oxygen species (ROS) and nitrogen species, bacterial attachment, motility and secretion.

### Validation of microarray expression data

**Figure 1:**
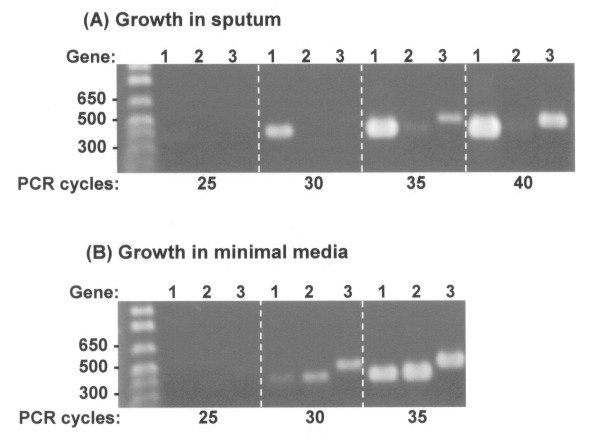
**Validation of microarray observed *B*. cenocepacia gene expression by semi-quantitative PCR**. The results of semi-quantitative RT-PCR on cDNA derived from sputum-grown *B. cenocepacia *(panel A) and from cells grown under control conditions (panel B) are shown. The products amplified after set numbers of PCR cycles (labelled below each panel) are shown for the following genes: 1, ferric reductase transmembrane gene (BCAL0270; up-regulated in sputum); 2, conserved hypothetical gene (BCAL1165; down-regulated in sputum); and 3, *phaC *(BCAL1861; control gene without altered expression). Molecular size markers are shown on the left hand side of each gel and the relevant size fragments indicated in bp.

We confirmed the microarray data by both semi-quantitative reverse transcriptase PCR and Real-Time PCR (RQ-PCR) designed to target five genes with altered expression and one control gene of constant expression (Table 2 and 3). The target genes selected were representative of: (i) a range of observed changes in expression (from 6- to 66-fold), and (ii) different functional classes of genes with altered expression (Table 3). Semi-quantitative PCR was initially applied to BCAL0270, encoding a putative ferric reductase transmembrane protein (36 × upregulated), and BCAL1165, a conserved hypothetical gene (6 × downregulated; RQ-PCR analysis of this gene was not possible due to limited sensitivity). Using pooled cDNA from the microarray experiments, in the sputum growth condition BCAL0270 was consistently amplified after 5 fewer cycles in comparison to the *phaC *(BCAL1861) house-keeping control gene while in contrast, BCAL1165 was not amplified until 35 cycles in comparison to the control gene BCAL1861 (Figure 1); this corroborated the microarray data showing that BCAL0270 was upregulated and BCAL1165 downregulated in sputum. During growth in BSM signals for BCAL270, BCAL1165 and the control *phaC *gene all appeared after 30 cycles (Figure 1) indicating similar levels of relative expression for all these genes in this growth condition. Although a signal for the control gene was absent in sputum yet present in BSM at 30 cycles of amplification (Figure 1), this probably resulted from less cDNA template being present in the sputum-recovered samples, as the microarray-observed gene expression had shown that the *phaC *control gene was stable in expression relative to all other genes for both sputum and BSM growth.

**Table 3:** Confirmation of *B. cenocepacia *gene expression by Real Time PCR

	**Real-time PCR fold change using delta delta C_T _method**			
	
**Gene expression determined from ** **(infection; clinical condition):**	**BCAL0270** (ferric reductase transmembrane component)	**BCAM2753** (*ohr *gene, organic hydroperoxide resistance protein)	**BCAL1107** (oxidoreductase)	**BCAM1676** (nitrite/sulfite reductase)
**Microarray dataset** (Sputum 1, 2, and 3; see below)	↑ 36	↑ 8	↑ 66	↑ 20
**Sputum 2** (*B. cenocepacia*; stable)	↑ 160	↑ 100	↑ 914	↑ 97
**Sputum 3** (*B. cenocepacia*; exacerbation)	↑ 65	↑ 40	↑ 299	↑ 4
**Sputum 4** (*B. cenocepacia*; stable)	↑ 25	↑ 22	↑ 235	-
**Sputum 5** (*P. aeruginosa*; exacerbation)	↑ 22	↑ 71	↑ 189	-
**Sputum 6** (*P. aeruginosa*; stable)	↑ 3	↑ 3	↑ 12	↑ 94

**Average for sputum growth**	**↑ 55**	**↑ 47**	**↑ 330**	**↑ 65**

***Fold change in minimal media with:***				

**No FeSO**_4_	↑ 2	-		
**0.1% H_2_O_2_**	-	↑ 5		
**0.15% H_2_O_2_**	-	↑ 3		
**0.002% t-butyl hydroperoxide**	-	↑ 2		

In addition to examining the cDNA used in the microarray analysis, RQ-PCR was then applied to fresh RNA extracted from *B. cenocepacia *J2315 grown in additional aliquots of sputum samples 2 and 3 that had been used for the microarray analysis, as well as samples from three additional CF patients (Table 3). This set of RNAs allowed gene expression to be tested in multiple population replicates, accounted for biological variability between samples, and enabled the calculation of mean values of gene alteration that were more representative of CF infection conditions. Expression analysis by RQ-PCR of four selected upregulated genes correlated well with the results observed by microarray analysis (Table 3).

Quantitative PCR demonstrated that each gene was consistently upregulated after *B. cenocepacia *growth in all five different sputum samples (Table 3). However, the fold change of expression varied considerably, with the most extreme case being the putative oxidoreductase encoding gene (BCAL1107) whose mean fold change across the five sputum samples was 5 times greater than had been observed for the three sputum samples examined using the microarrays (Table 3). Although the differences in upregulation were substantial, all were similar to or above that seen in the microarray analysis except for expression of BCAL0270, BCAM2753, and BCAL1107 observed after *B. cenocepacia *growth in sputum 6 (Table 3). Each of the latter genes were still upregulated in sputum 6 compared to the control, however, the values were 12, 2.5 and 5.5 less, respectively, than those measured from the microarray dataset.

The excellent correlation between the microarray data and RQ-PCR demonstrated that the *B. cenocepacia *custom microarray was performing very well and also illustrated the importance of using of the three biological replicates (3 CF individuals) to provide an unbiased set of sputum-growth microarray data. RQ-PCR data related to two selected genes (BCAL0270 and BCAM2753) will be discussed further in the relevant sections describing the functional classes of genes with altered expression during *B. cenocepacia *growth in sputum.

### *B. cenocepacia *antimicrobial resistance

CF patients are typically treated with a combination of antibiotics including beta-lactams, macrolides, tetracyclines, aminoglycosides and polymyxins. At the time of sputum collection each patient was receiving antibiotics as follows: patient 1 was on long-term flucloxacillin and azithromycin and had just started a course aztreonam and tobramycin at the time the specimen was provided; patient 2 was receiving a course of azithromycin and minocycline, while patient 3 was on long-term azithromycin therapy with the additional prescription of ceftazidime, fluconazole and gentamicin. Hence the identification of antibiotic resistance genes with altered expression was expected due to traces of antibiotics being present in the sputum samples as a result of daily therapy.

Antibiotic efflux is known to be a primary resistance mechanism in Bcc bacteria [2]. After growth of *B. cenocepacia *in sputum, we detected gene expression changes in three of the multiple resistance-nodulation-cell division (RND) efflux operons [19]: BCAL1675 and BCAM1947 were upregulated while BCAL1813 was downregulated (Table 4). The *ceo *operon is arguably the most well characterised RND pump encoding cluster in *B. cenocepacia *(corresponding to genes BCAM2549-2552, 2554) [20]. We did not detect any expression changes in genes from the *ceo *cluster, correlating to a lack of chloramphenicol, trimethoprim or ciprofloxacin therapy in the patients examined. Significantly altered expression was also found in five more efflux transporter systems which did not belong to RND family (upregulated pumps were: BCAM0200, BCAM0791/792; downregulated pumps were BCAL1510, BCAL3511/514 and BCAS0015; Table 4).

**Table 4:** Major groups of *B. cenocepacia *genes with altered gene expression in sputum

**Gene number:**	**Fold change in expression**	**Putative gene function:**
***Antimicrobial resistance***		

**upregulated**		
BCAL1675	2.67	multidrug efflux system transporter protein AmrB
BCAM0200	2.20	efflux system transport protein
BCAM0791	2.11	Major Facilitator Superfamily protein
BCAM0792	2.45	efflux system transport protein
BCAM1362	2.06	putative penicillin-binding protein
BCAM1947	3.76	putative quinoxaline efflux system transport protein
BCAS0034	19.84	metallo-beta-lactamase superfamily protein

**downregulated**		
BCAL0704	2.61	D-alanyl-D-alanine carboxypeptidase (penicillin-binding protein precursor)
BCAL1510	2.72	putative multidrug resistance transporter protein
BCAL1813	2.37	multidrug efflux system outer membrane protein
BCAL2832	2.07	D-alanyl-D-alanine carboxypeptidase/endopeptidase (penicillin-binding protein precursor)
BCAL3511	2.08	multidrug resistance transporter protein
BCAL3514	2.11	outer membrane efflux protein
BCAS0015	2.09	efflux system transport protein

***Iron uptake & metabolism***		

**upregulated**		
BCAL0270	35.59	ferric reductase-like transmembrane component
BCAL0273	8.23	protein CyaY
BCAM2231	2.86	transcriptional regulator PchR
BCAM2232	2.11	putative pyochelin biosynthetic protein PchD

**downregulated**		
BCAL2812	2.59	putative Fur family transcriptional regulator
BCAL3201	2.59	putative TolR-related protein
BCAL3203	2.18	putative periplasmic TolB protein
BCAL3204	2.67	putative OmpA family lipoprotein

***Protection against reactive oxygen and nitrogen species***		

**upregulated**		
BCAL1766	2.23	OsmC-like protein
BCAM1676	19.99	putative nitrite/sulfite reductase
BCAM1677	26.75	conserved hypothetical protein
BCAM2753	8.00	putative organic hydroperoxide resistance protein, *ohr *gene

**Downregulated (none)**		

***Motility and adherence***		

**upregulated**		
BCAL0124	2.02	flagellar regulon master regulator subunit FlhD
BCAL0525	2.17	flagellar M-ring protein FliF
BCAL0527	2.34	flagellar protein FliS
BCAL3506	2.58	flagellar motor switch protein FliM
BCAM0987	2.69	flagellar hook protein 2 FlgE2
BCAM2143	2.33	cable pilus associated adhesin protein
BCAS0299	3.55	flp type pilus subunit

**downregulated**		
BCAL3504	3.31	flagellar protein FliO
BCAM2758	2.35	two-component regulatory system, sensor kinase protein
BCAM2759	3.73	putative minor pilin and initiator
BCAM2761	2.53	giant cable pilus
BCAM2762	2.81	giant cable pilus chaperone protein
BCAS0301	2.03	putative flp type pilus leader peptidase

***Secretion***		

**upregulated**		
BCAL0849	3.39	metallo peptidase, subfamily M48B
BCAL3517	2.36	type II secretion system protein L
BCAM2040	2.98	type III secretion system protein
BCAM2042	2.16	type III secretion system protein
BCAS0196	3.25	putative polygalacturonase
BCAS0409	6.36	zinc metalloprotease ZmpA

**downregulated**		
BCAL3515	2.62	type II secretion system protein N
BCAL3521	3.19	type II secretion system protein I
BCAL3522	2.21	type II secretion system protein H
BCAM0240	2.47	N-acylhomoserine lactone dependent regulatory protein

***Selected gene clusters***		

**upregulated**		
BCAL0269	20.82	putative oxidoreductase
BCAL0270	35.59	ferric reductase-like transmembrane component
		
BCAL1106	90.32	cytochrome b561 family protein
BCAL1107	65.79	putative oxidoreductase
		
BCAL1153	3.30	putative salicylaldehyde/benzaldehyde dehydrogenase
BCAL1156	2.02	putative 4-hydroxybenzoate transporter
BCAL1157	2.51	putative monooxygenase
		
BCAM1676	19.99	putative nitrite/sulfite reductase
BCAM1677	26.75	conserved hypothetical protein
		
BCAM2749	31.39	carboxymuconolactone decarboxylase family protein
BCAM2750	14.06	putative exported protein
BCAM2751	7.13	LysR family regulatory protein
BCAM2752	12.35	NAD dependent epimerase/dehydratase family protein
BCAM2753	8.00	putative organic hydroperoxide resistance protein
BCAM2754	10.46	putative ketoreductase
**downregulated**		
BCAL1130	2.10	conserved hypothetical protein
BCAL1161	2.46	conserved hypothetical protein
BCAL1162	2.30	TetR family regulatory protein
BCAL1165	5.59	conserved hypothetical protein
BCAL1166	4.07	conserved hypothetical protein
BCAL1168	3.06	conserved hypothetical protein
BCAL1169	2.05	conserved hypothetical protein (pseudogene)
		
BCAL3104	8.00	urease gamma subunit
BCAL3105	2.22	urease beta subunit
BCAL3106	2.34	urease alpha subunit
BCAL3109	2.42	urease accessory protein
		
BCAM0066	5.13	putative lipoprotein
BCAM0067	14.47	putative short chain dehydrogenase
BCAM0068	11.74	Major Facilitator Superfamily protein
BCAM0069	21.83	conserved hypothetical protein
BCAM0070	15.46	putative hydrolase
BCAM0071	11.11	puatative mandelate racemase lactonizing enzyme
BCAM0072	16.58	putative thiamine pyrophosphate enzyme
BCAM0073	6.85	hypothetical protein
BCAM0074	9.43	conserved hypothetical protein

Enzymatic inactivation is another mechanism of antibiotic resistance that is intrinsic to *B. cenocepacia *especially with regard to chromosomally encoded betalactamases [2]. However, none of the 4 annotated betalactamases (BCAM0393, BCAM1179, BCAM2165 and BCAS0156) in the genome were altered in expression, however, a 20-fold change in BCAS0034 was observed. This chromosome 3 encoded gene possesses a conserved metallo-betalactamase domain but whether the encoded protein may act as functional betalactamase remains to be determined. The potential effects of beta-lactam compounds being present in the sputum examined was also indicated by expression changes observed in genes encoding penicillin binding proteins, of which BCAL0704 and BCAL2832 were downregulated, while BCAM1362 was upregulated (Table 4).

### Iron uptake and metabolism

Iron is an essential element required by microorganisms for many biological processes, yet its supply in a form of free, non-bound iron, is limited in the host environment. Pathogenic bacteria have evolved various iron acquisition mechanisms for the utilization of extracellular iron sources, with siderophore secretion and uptake being one of the most effective systems. *B. cenocepacia *produces several types of siderophore including ornibactin, pyochelin and salicylate, although the salicylate might function as a pyochelin precursor rather than a siderophore per se [21]. During the growth in sputum *B. cenocepacia *activated two genes involved in biosynthesis of salicylate/pyochelin (*pchR*; BCAM2231 and *pchD*; BCAM2232) and suppressed the expression of a transcriptional repressor Fur, the global iron regulator (BCAL2812). Interestingly, the gene cluster encoding biosynthesis of another *B. cenocepacia *siderophore, ornibactin (BCAL1688-1702), was not activated during sputum growth; this siderophore has, however, been shown to be important for the pathogenesis in rat model of chronic lung infection [22]. The increased demand of the bacterium for iron was also reflected by immense over-expression (> 35×) of a membrane ferric reductase (BCAL0270) which catalyzes reduction of siderophore-bound ferric ions to soluble ferrous ions.

The Tol system in *P*. *aeruginosa *is also known to mediate transport of iron-containing siderophores into bacterial cells and under iron-restricted conditions at the late log stage of growth, the *P. aeruginosa tol *genes are known to be downregulated [23]. Three *B. cenocepacia *genes homologous to the *tol *system (BCAL3201, BCAL3203-3204) were found to be downregulated in sputum suggesting that the growth environment was iron-restricted. Once in cytoplasm, iron can be sequestered in storage proteins such as bacterioferritin or iron-sulphur proteins. We detected significantly higher expression of *cyaY *gene (BCAL0273, 8 fold upregulation; Table 4) which is a bacterial ortholog of a protein frataxin, an iron donor for assembly of Fe-S clusters in eukaryotes [24].

Although the infected CF lung has traditionally been considered an iron-deficient environment, this dogma has been the subject of considerable research and debate [25]. The significant expression changes in *B. cenocepacia *genes involved in the iron cycle implied that the sputum-based growth model we examined represented an iron-depleted condition. The overall iron concentration in CF sputum, approximately 3 × 10 ^-5 ^M [26] is high from a bacterial growth standpoint. The total iron concentration in our 10% mixtures of sputum 2 and 3, was 3.6 × 10^-5 ^M (1.98 ppm) and 3.5 × 10^-5 ^M (1.96 ppm), respectively, and was comparable to the control basal salts medium at 3.7 × 10^-5 ^M (2.1 ppm). However, the total iron measured in sputum is composed of both ionic forms, of which only the soluble ferrous ions can be readily utilized by bacteria without using siderophores. Hence, in our experimental model the ferrous form of iron was likely to have been lower in the 10% sputum medium, than in the control BSM which contained solely soluble ferrous ions. The microarray data and RQ-PCR results on additional sputum samples had shown substantial up-regulation of a ferric reductase membrane protein gene (BCAL0270), ranging from 3 to 160 fold increases (Table 3), suggesting that *B. cenocepacia *gene expression was being altered due to iron limitation. To mimic the assumed iron-depleted condition and evaluate its linkage to the iron-regulated gene expression, *B. cenocepacia *J2315 was grown in minimal media BSM with no iron salts added (4.1 × 10^-6 ^M available iron; 0.2 ppm) and the expression of BCAL0270 quantitated as an iron marker gene. Growth of *B. cenocepacia *in the low iron media resulted in a 2 fold up-regulation of BCAL0270, corroborating the assumption that its expression in sputum was being activated by a reduction in iron concentration (Table 3).

### Protection against reactive oxygen and nitrogen species

Bacteria possess multiple mechanisms to protect themselves from toxic reactive oxygen species such as hydrogen peroxide, hydroxyl radicals, superoxide and organic hydroperoxides. Bacterial respiration and metabolism is an additional endogenous source of ROS and during growth in sputum this metabolic reservoir appeared to have increased leading to extreme up-regulation in two oxidoreductases (BCAL0269, 20-fold; BCAL1107, 65-fold; Additional File 1) and cytochrome b (BCAL1106; > 90 fold; Additional File 1). Since CF patients experience chronic lung inflammation their sputum is also reservoir of harmful ROS compounds. Although we expected that a large number of ROS defence mechanisms to be activated, only two genes, both previously uncharaterized in *B. cenocepacia*, were significantly upregulated during growth in sputum. An 8-fold increase in expression of the hydroperoxide resistance gene, *ohr *(BCAM2753; Table 4) was observed; this gene is known to be inducible by organic peroxides [27] and to lesser extent by hydrogen peroxide [28]. The second, slightly upregulated (2.2-fold) gene was *osmC *(BCAL1766) that is also involved in defence against oxidative stress [29].

The significant fold change in *ohr *expression suggested that the induction of bacterial defence against ROS had taken place, despite the fact that antioxidant encoding genes [30] such as catalases (KatA and KatB), alkyl hydroperoxide reductases (AhpC) and superoxide dismutases (SodB and SodC), remained unaffected. To validate the oxidative stress model formulated from the microarray results, we performed further set of growth experiments where *B. cenocepacia *J2315 cells were exposed to sub-inhibitory levels of ROS (0.1% H_2_O_2_, 0.15% H_2_O_2 _and 0.002% tert-butyl hydroperoxide, a member of organic hydroperoxides family). After RNA extraction, the expression of *ohr *was then determined by RQ-PCR analysis. The quantitative PCR results showed activation of *ohr *in all test conditions, with the greatest response occurring after exposure to H_2_O_2 _(Table 3), confirming the involvement of *ohr *in mediating *B. cenocepacia *response to both inorganic and organic forms of peroxides.

In addition to oxidative stress, infecting bacteria are threatened by nitrosative stress coming from reactive nitrogen species (RNS) such as nitric oxide radicals [31]. Although the mechanisms of enzymatic detoxification of RNS are not as well characterized as the responses to ROS, there is evidence that nitrite reductase enzyme may play an important role [32]. Growth of *B. cenocepacia *in sputum triggered very high expression of a nitrite/sulfite reductase (BCAM1676; 20-fold) and its neighbouring gene of unknown function (BCAM1677; 26-fold); a potential role in infection for these two genes has not been previously implicated. Also since *B. cepacia *complex bacteria are well known to be very resistant to the neutrophil-mediated non-oxidative killing [33], the significant upregulation of this nitrite/sulfite reductase pathway may form the basis for their resistant phenotype.

### Motility and adherence

*B. cenocepacia *are motile bacteria and possess one or more flagella. In addition to mediating motility, the flagellum may also act as an adhesin establishing a primary contact with epithelial cells and playing a central role in establishing the initial phases of host colonization [2]. *B. cenocepacia *flagellum-mediated motility has also been shown to enable it to invade host cells [34]. Over 40 genes, which are distributed in several discrete clusters across the genome and whose expression is arranged hierarchically, contribute to the synthesis and assembly of the whole flagellum.

Growth in sputum increased the expression of flagellar structural genes (basal body MS-ring gene *fliF*; BCAL0525, main flagellin chaperone gene *fliS*; BCAL0527, C-ring gene *fliM*; BCAL3506, hook-protein gene *flgE*; BCAM0987; Table 4) as well as of one regulatory gene (*flhD*; BCAL0124; Table 4). In two of the three patients sputa examined, the major structural component, flagellin (*fliC*; BCAL0114), was also found to be overexpressed (with a 3-fold change in expression), however, the gene was excluded from the final gene list because of the filtering criteria that was applied across the entire dataset (see Methods and Materials). Only one gene associated with assembly of the flagellum was downregulated, *fliO *(BCAL3504; Table 4); this gene is thought to be part of the type III secretion system involved in the flagellar protein export [35]. Why this particular gene exhibited lower expression was not clear since other components of the secretion system (i.e., *fliH*; BCAL0523, *fliI*; BCAL0522, *fliP*; BCAL3503, *fliQ*; BCAL3502 and *fliR*; BCAL3501) did not change their level of expression and the whole system is believed to be necessary for transmembrane transport of the synthesized flagellar components.

The changes in expression of adhesins were not limited to flagellum, but also occurred in pili encoding genes. The gene for structural units of recently described type IV pili (*flp*; BCAS0299) [36] was found to be significantly upregulated (Table 4), although its leader peptidase exhibited slightly decreased expression (BCAS0301; Table 4). Other fimbriae affected by growth in sputum were the cable pili which are exclusively expressed by strains of ET12 clonal complex [37]. Nearly the entire cable pilus gene cluster was significantly downregulated (*cblS*, BCAM2758; *cblD*, BCAM2759; *cblA*, BCAM2761 and *cblB*, BCAM2762; Table 4) suggesting that during growth in CF sputum these surface structures are not essential. In contrast, the cable pilus adherence-mediating 22 kDa adhesion protein (*adhA*; BCAM2143) [38], was found to be upregulated (Table 4).

### Secretion

Bacteria use various secretory pathways to translocate exoproducts and proteins that form their external cellular structures through the membranes. Two of them, type II and type III secretion systems, exhibited altered expression in their component genes during the growth of *B. cenocepacia *in sputum. The majority of type II secretion genes were downregulated (BCAL3515, BCAL3521-3522; Table 4) with the only exception was being upregulation of BCAL3517 (Table 4). Two components of the *B. cenocepacia *type III secretion system showed increased expression (BCAM2040 and BCAM2042; Table 4). Type II secretion systems have an evolutionary relationship with the Type IV pilus [39], a component of which, BCAS0299, was also upregulated (Table 4); Type III secretion systems share homology with the flagella gene systems [40] which were also upregulated in *B. cenocepacia*. The upregulation of all the latter systems suggest that there may be a common regulatory mechanism present that controls their transcription in *B. cenocepacia*.

Many bacterial exoproducts are known to play important roles in infection and two proteases with altered gene expression were notable in the context of *B. cenocepacia *virulence in CF. The ZmpA metalloprotease is a well characterised Bcc virulence factor which is known to play a role during infection when examined rat chronic lung infection model [41]. The gene encoding ZmpA, BCAS0409, was upregulated 6-fold (Table 4) during growth in sputum, corroborating the data from animal studies that this is a core *B. cenocepacia *virulence factor. A second, as yet uncharacterised protease, BCAL0849 was also upregulated 3-fold (Table 4) suggesting it may also play a role during growth in sputum. However, no alteration in expression of ZmpB (BCAM2307), another well characterised Bcc protease [42], was detected.

### Gene clusters

In addition to functional classification, we identified several clusters of genes where CDSs in close vicinity to each other demonstrated an altered expression (Table 4). These putative operons contained two or more genes with remarkably high values transcriptional fold change (2 to 90 fold) which further underlined a very likely co-regulation of their transcription. Two under expressed gene clusters represented well defined operons: the cable pilus operon (as described above; Table 4) and a cluster of urease structural protein genes (significantly downregulated were BCAL3104-6 and BCAL3109; Table 4). The remaining putatively co-regulated gene clusters comprised groups of genes that were either hypothetical or their functional classifications were rather general in nature. Out of these groups, a complex of nine consecutive hypothetical genes which all showed a 5–22 fold decrease in expression (BCAM0066-0074) was interesting. A similar cluster, also exhibiting coordinated expression of all genes but this time significant upregulation, comprised genes BCAM2749-2754 including the above mentioned hydroperoxide resistance gene *ohr *(Table 4). In the absence of functional data on these genes the physiological significance of these findings remain to be determined, however, they do illustrate the ability of transcriptomic analysis to reveal novel genes implicated in bacterial pathogenesis.

Several pairs of upregulated genes were identified which featured extreme changes in their expression. The highest values in the dataset were detected for the pair BCAL1106-1107 (cytochrome b and oxidoreductase; described above, Table 4). Other pairs of high expression were BCAL0269-0270 (oxidoreductase and ferric reductase; Table 4) and BCAM1676-1677 (nitrite/sulphite reductase and conserved hypothetical protein; Table 4).

A feature of the genomic content and evolution of *Burkholderia *bacteria is the presence of a large amount (approximately 10%) of recently acquired DNA in the form of genomic islands [2,12]. However, very few of the differentially expressed CDSs were found to reside within *B. cenocepacia *J2315 genomic islands. This suggested that the factors necessary for pathoadaptation to cystic fibrosis and early growth in sputum were more inherent *B. cenocepacia *traits and were not recently acquired via mobile genetic elements. Only one island on the largest chromosome (designated BcenGI5; M. Holden, unpublished data), contained significant number of differentially expressed genes and this comprised a mixture of both up- and down-regulated genes. Three of the upregulated CDSs (BCAL1153, 1156, 1157) comprise part of a putative hydroxybenzoate catabolism cluster, and includes a salicylaldehyde dehydrogenase (BCAL1153), which catalyses the conversion of salicylaldehyde to salicylate. Although it appears that the salicylaldehyde dehydrogenase is part of a horizontally acquired xenobiotic degradation pathway, this enzyme may also have an additional role in the iron metabolism of J2315 as discussed above. Seven genes within the island were downregulated (BCAL1130, 1161–1162, 1165–1166 and 1168–1169; Table 4) and were of no known function. Transcriptomic analysis also demonstrated that apart from downregulation of the quorum-sensing regulator, *cciIR *(Table 4), no other gene on the Cenocepacia Pathogenicity Island (cci) [43] was altered in expression, suggesting that this island implicated in chronic lung infection is not extensively involved in rapid growth in CF sputum.

## Discussion

Our study is the first full transcriptomic examination of a CF pathogen from the *B. cepacia *complex. We have extensively validated a custom microarray designed to the *B. cenocepacia *J2315 genome, demonstrating that it produces reproducible and quantitative results, as well as testing and confirming the growth models of iron-restriction and oxidative-stress formulated from the genomic expression profiles. Understanding how infecting bacteria colonize, grow and survive within sputum is fundamental to respiratory pathogenesis in CF, and by examining six individuals in this study we also considerably expanded on previous work examining the transcriptomic response of the major CF pathogen, *P. aeruginosa *(a study where sputum from two CF patients was examined [13]). Many of the gene pathways activated by *B. cenocepacia *in response to growth in sputum correlated to known virulence mechanisms [2]; eg. antibiotic efflux and degradation, iron metabolism, resistance to host defenses and motility), however, what was particularly gratifying about the microarray data was its ability to identify novel genes involved in these functions and to highlight unexpected trends in *B. cenocepacia *gene expression after growth in sputum. As a method to examine virulence in an opportunistic pathogen with a large genome such as *B. cenocepacia*, our choice to use a microarray-based experimental strategy was fully validated.

The inability to clear Bcc bacteria from the CF lung with antibiotics frequently leads to chronic respiratory infection and the emergence of pan-resistant strains that require combination therapy to suppress [44]. Growth of *B. cenocepacia *in sputum revealed that 8 efflux pumps systems (3 that were members of the RND family) were altered in expression (4 up- and 4 down-regulated; Table 4). Thirteen putative multidrug efflux pumps of RND family have been characterised in *B. cenocepacia *J2315 genome; four of them have been shown to be transcriptionally active during the growth in Luria-Bertani medium, although their mode of expression (constitutive or regulated) was not elucidated [19]. Our transcriptomic data indicated that three of these RND pumps are under transcriptional regulation (BCAL1675, BCAL1813 and BCAM1947; Table 4) and not constitutively expressed in *B. cenocepacia*. These three RND systems and the five additional efflux transport systems highlighted by the microarray analysis are worthy of further study in relation to their role in growth and antibiotic resistance. Looking for inhibitors of these systems may improve the efficacy of current antibiotics as well as weaken the ability for *B. cenocepacia *to grow in sputum.

The *B. cenocepacia ceo *efflux system [20] was not activated in any of the sputum samples, an observation that correlated to the lack chloramphenicol, trimethoprim or ciprofloxacin therapy. The *ceo *pump is also known to actively efflux salicylate [20], whose biosynthetic pathway was upregulated in sputum along with several other iron-acquisition systems. Nair et al. [20] hypothesized that there was a redundancy in salicylate transport systems since Bcc strains lacking a complete set of *ceo *genes were detected in their study, an observation that appears to be supported by the lack of *ceo *expression yet siderophore overexpression seen in the microarray data. Candidate salicylate transporters may constitute the other sputum-activated RND efflux pumps (BCAL1675 and BCAM1947; Table 4).

It is also possible that the observed increase in RND pump expression (see above) may have also been induced by low iron concentrations rather than by presence of antibiotics per se. A link between iron, siderophore secretion and RND pump activity has been described in *P. aeruginosa *where mutants lacking *mexA *or *mexB *RND efflux pump encoding genes were not able to grow on iron-deficient medium [45]. Factors involved in iron acquisition such as siderophores and their uptake mechanism are known to be important for *B. cenocepacia *virulence [21]. However, the microarray analysis also revealed potentially important roles for a ferric reductase in iron uptake and the frataxin-like *cyaY *gene in iron storage and the formation of Fe-S clusters. A link between CyaY and Fe-S protein biosynthesis has been recently proposed for prokaryotes [24]. Based on such evidence, we suggest that Fe-S cluster may play a primary role in iron storage during growth of *B. cenocepacia *in sputum.

Recently, Kohanski et al. [46] proposed a cascade of biochemical events which take place in bacterial cells when exposed to bactericidal antibiotics. The pathway, which is characterized by production of hydroxyl radicals via Fenton chemistry, may represent an ultimate killing mechanism of bactericidal antibiotics. In the Kohanski model, the formation of ROS occurs via oxidation of ferrous ions whose key intracellular source is thought to be iron-sulphur clusters [46]. Our study revealed significant changes in activity of several genes which may be pieces of the same puzzle in *B. cenocepacia *and represent the bacterial protective response against hydroxyl radicals: *cyaY *(a frataxin-like gene; BCAL0273), *ohr *(hydroperoxide resistance gene; BCAM2753) and iron acquisition genes (siderophores, salicylate biosynthesis and membrane ferric reductase). Since CF patients are on constant antimicrobial therapy, the large amounts of residual antibiotics in sputum may stimulate oxidation of free ferrous ions in the Fenton reaction, hence triggering an imbalance in intracellular iron distribution which results in the activation of the iron cycle genes we have observed in *B. cenocepacia*.

In addition to identifying potentially new *B. cenocepacia *virulence mechanisms, the transcriptomic analysis also revealed interesting trends in the expression of known virulence factors. For example, the upregulation of flagellar genes observed in *B. cenocepacia *contrasted markedly with the results of *P. aeruginosa *transcriptomic analysis, where the expression of flagella was repressed during the incubation in CF sputum [13]. While adaptation to a non-motile phenotype is a well-characterised trait of *P. aeruginosa *during chronic CF infection [47], this phenomenon has not been observed in Bcc bacteria. The upregulation of *B. cenocepacia *flagellar apparatus genes suggests that motility may allow this CF pathogen to maintain a more invasive phenotype during infection. Maintaining motility may also correlate to the fact that *B. cenocepacia *and other Bcc bacteria retain their ability to cause a septic, invasive, "cepacia syndrome" at any point during chronic CF infection [5,6].

Another example of an unexpected expression finding was the lack of activation of the structural components of cable pilus operon of *B. cenocepacia *J2315. Studies on a *B. cenocepacia *cable pilus knockout mutant showed that it was not altered in its capacity to bind lung epithelial cells [48], which taken together with our transcriptomic data, this suggests that the complete cable pilus may not be as important colonization surface structure as initially presumed [37]. However, we did find that the pilus-associated adhesin gene, *adhA *[38], was activated in sputum suggesting it is important for growth. In absence of cable pilus expression this finding also indicates that the AdhA adhesin may be incorporated onto other *B. cenocepacia *surface components.

Quorum sensing, the bacterial cell-to-cell communication system, controls many virulence genes functions in *B. cenocepacia *[2,49,50]. We did not detect expression changes in three of the characterized *B. cenocepacia *quorum sensing genes (i.e. N-acylhomoserine lactone synthases *cepI *and *cciI*, and a response regulator *cepR *[43,49]). This may have due to the fact that *B. cenocepacia *was only allowed to grow to mid-log phase prior to microarray analysis, and quorum sensing activity only normally appears at later stages of growth. Limited signs of quorum sensing activity were apparent from the overexpression of polygalacturonase gene (BCAS0196), the *zmpA *metalloprotease and motility-associated flagellar genes, which are all under its control [2,49,50]. The only quorum sensing gene exhibiting altered expression (a 2.5-fold under-expression) was *cciR *(BCAM0240; Table 4) which encodes a quorum sensing transcriptional regulator known to be an autorepressor [49].

## Conclusion

Studying opportunistic bacterial pathogens with large genomes, poor genetic tractability and limited models of infection is not straightforward. Our transcriptomic analysis of *B. cenocepacia *growth in a sputum-based model has provided a detailed snapshot of components of the J2315 genome that may promote colonisation and early growth in the CF lung. Transcriptomic analysis corroborated previous studies that had shown crucial roles for iron uptake and proteases in *B. cenocepacia *virulence. It also demonstrated that upregulation of efflux proteins and genes involved in antimicrobial stress are important. However, from the multiple genetic pathways encoding the latter phenotypic traits, microarray analysis demonstrated that previously uncharacterised *B. cenocepacia *genes such as a transmembrane ferric reductase (BCAL0270), protease (BCAL0849), organic hydroperoxide (BCAM2753), oxidoreductase (BCAL1107) and nitrite/sulfite reductase (BCAM1676), are significantly upregulated in sputum. In contrast to the pathogenesis of *P. aeruginosa *in CF lung infection, the expression of genes for motility and flagellar biosynthesis were upregulated, allowing *B. cenocepacia *to maintain a potentially invasive phenotype characteristic of cepacia syndrome. Virulence factors such as the structural cable pilus and genes on the cci were not upregulated during sputum growth suggesting they do not play an important role at the initial stage of infection we modelled. Overall, our successful transcriptomic analysis has revealed several new virulence *B. cenocepacia *virulence mechanisms worthy of further study and provided a reference set of gene expression data for growth of this pathogen in CF sputum.

## Competing interests

The authors declare that they have no competing interests.

## Authors' contributions

PD performed the microarray analysis, all experimental procedures associated with the transcriptomic study, and wrote the first draft of the manuscript. MTGH provided detailed correlation of the microarray data to the genome of *B. cenocepacia*. ZG provided assistance with the design of the microarray experiments and controls required to validate the performance of the arrays. AMJ and IK provided the clinical input concerning the cystic fibrosis individuals examined. RTG designed the *B. cenocepacia *custom microarray. EM conceived and planned the original study. PD, MTGH and EM wrote the paper and all authors have read the final manuscript.

## Pre-publication history

The pre-publication history for this paper can be accessed here:



## Supplementary Material

Additional file 1 Complete list of *B. cenocepacia *genes significantly upregulated after growth in CF sputum. *B. cenocepacia *genes that were significantly upregulated after growth in CF sputum are listed in a Microsoft Excel spreadsheet. The fields listed within the spreadsheet are: (i) the systematic gene identification number, (ii) the fold change in expression observed, (iii) its statistical significance in terms of a p value, (iv) the annotation and putative function of the genes and (v) the type of feature (CDS = coding sequence; tRNA; RNA; or intergenic region). Click here for file

Additional file 2 Complete list of *B. cenocepacia *genes significantly downregulated after growth in CF sputum. *B. cenocepacia *genes that were significantly downregulated after growth in CF sputum are listed in a Microsoft Excel spreadsheet. The fields listed within the spreadsheet are: (i) the systematic gene identification number, (ii) the fold change in expression observed, (iii) its statistical significance in terms of a p value, (iv) the annotation and putative function of the genes and (v) the type of feature (CDS = coding sequence; tRNA; RNA; or intergenic region). Click here for file
